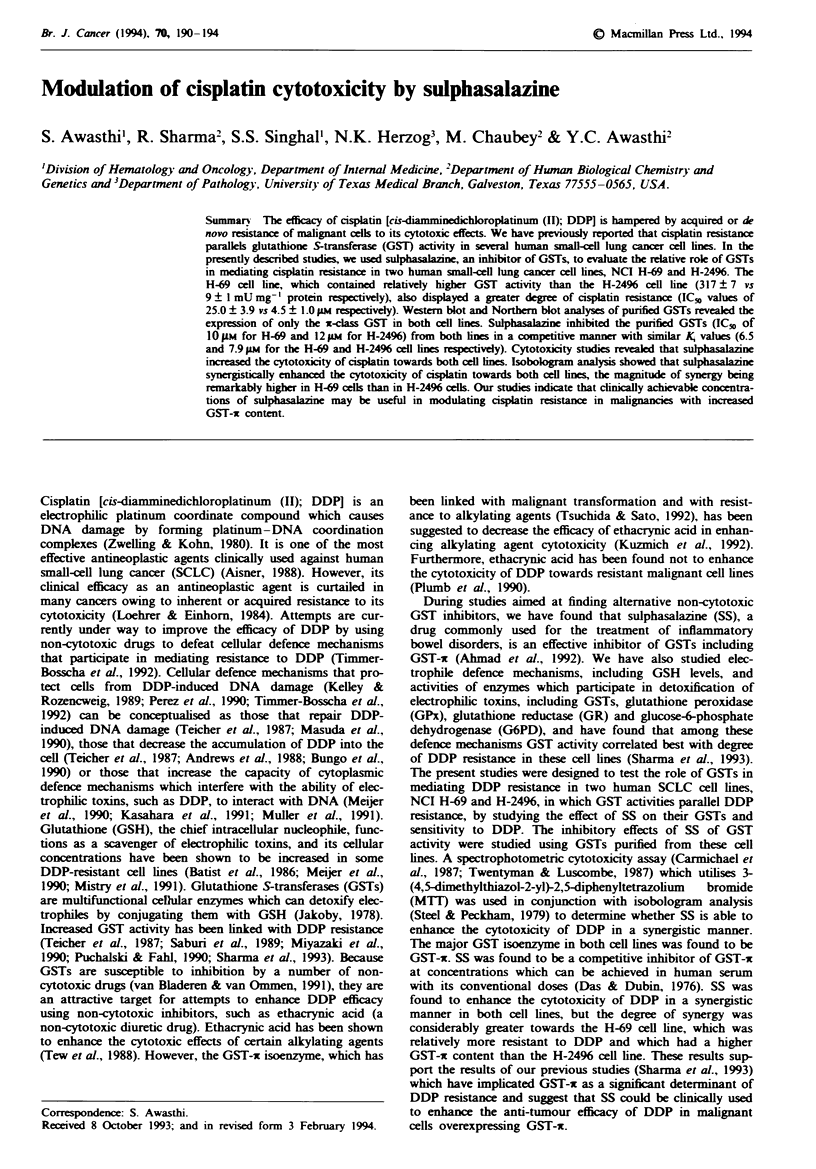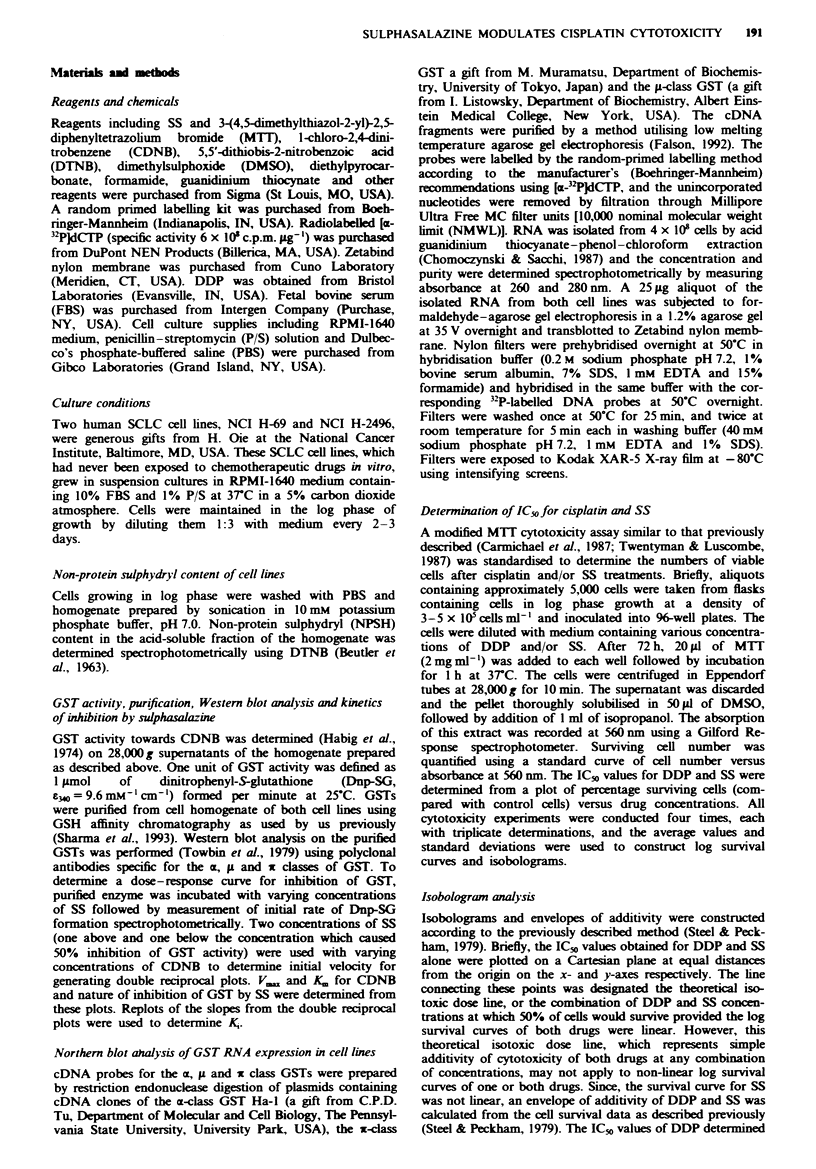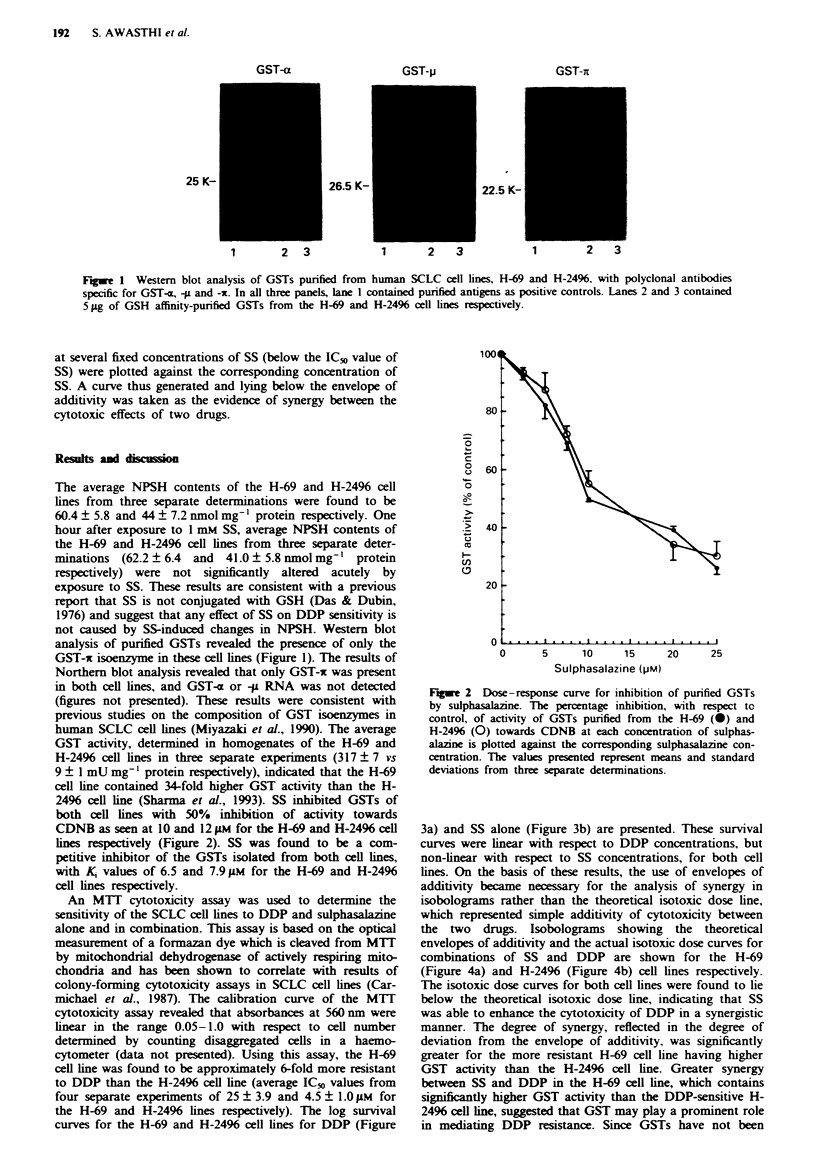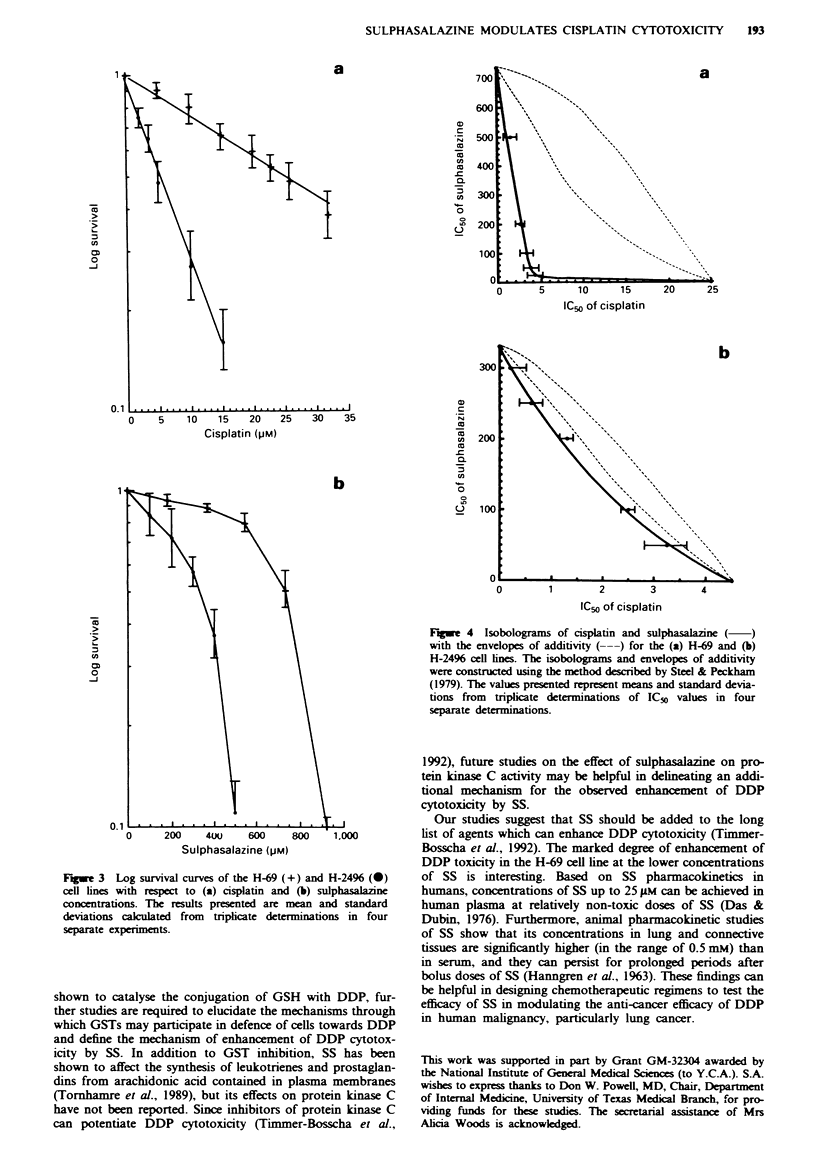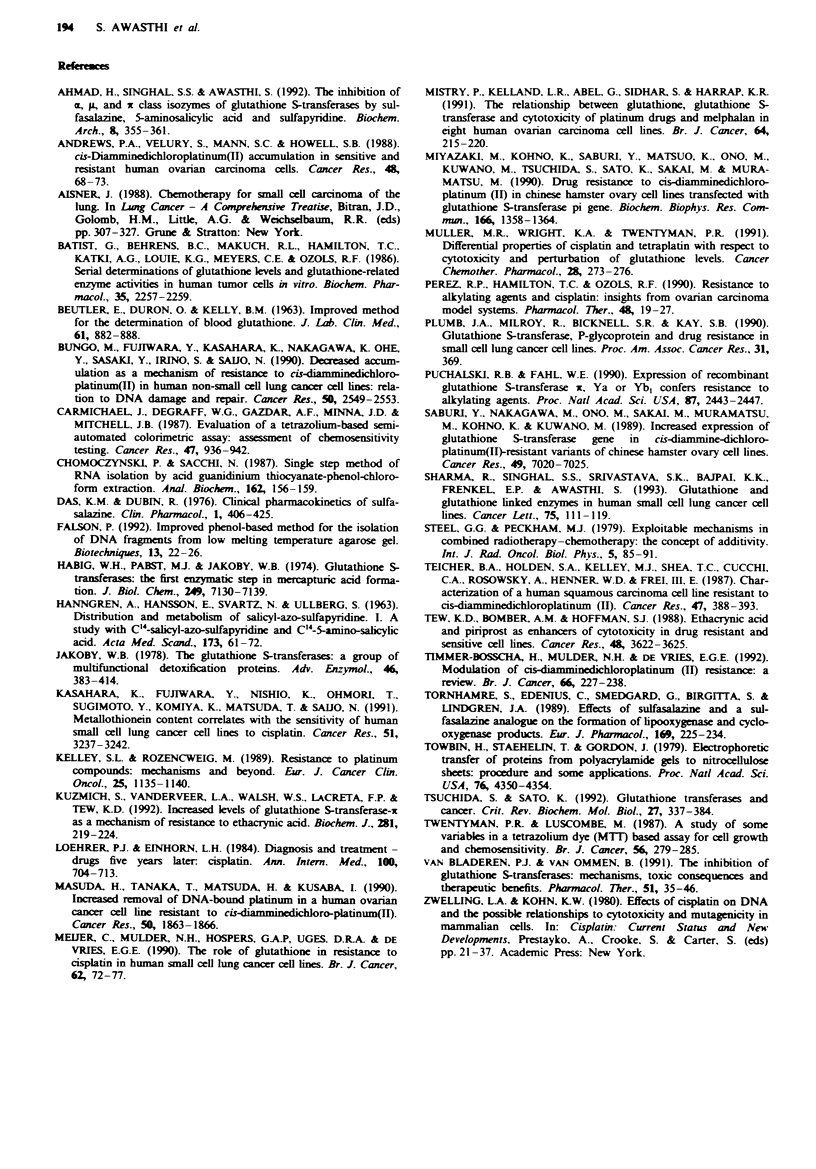# Modulation of cisplatin cytotoxicity by sulphasalazine.

**DOI:** 10.1038/bjc.1994.278

**Published:** 1994-08

**Authors:** S. Awasthi, R. Sharma, S. S. Singhal, N. K. Herzog, M. Chaubey, Y. C. Awasthi

**Affiliations:** Department of Internal Medicine, University of Texas Medical Branch, Galveston 77555-0565.

## Abstract

**Images:**


Br. J. Cancer (1994), 70, 190-194 © Macmillan Press Ltd., 1994

### Modulation of cisplatin cytotoxicity by sulphasalazine

S. Awasthi[1], R. Sharma[2], S.S. Singhal[1], N.K. Herzog[3], M. Chaubey[2] & Y.C. Awasthi[2]

[1]*Division of Hematology and Oncology, Department of Internal Medicine,* [2]*Department of Human Biological Chemistry and Genetics and* [3]*Department of Pathology, University of Texas Medical Branch, Galveston, Texas 77555-0565, USA.*

**Summary** The efficacy of cisplatin [*cis*-diamminedichloroplatinum (II); DDP] is hampered by acquired or *de novo* resistance of malignant cells to its cytotoxic effects. We have previously reported that cisplatin resistance parallels glutathione *S*-transferase (GST) activity in several human small-cell lung cancer cell lines. In the presently described studies, we used sulphasalazine, an inhibitor of GSTs, to evaluate the relative role of GSTs in mediating cisplatin resistance in two human small-cell lung cancer cell lines, NCI H-69 and H-2496. The H-69 cell line, which contained relatively higher GST activity than the H-2496 cell line ($317 \pm 7$ *vs* $9 \pm 1$ mU mg$^{-1}$ protein respectively), also displayed a greater degree of cisplatin resistance ($IC_{50}$ values of $25.0 \pm 3.9$ *vs* $4.5 \pm 1.0$ μM respectively). Western blot and Northern blot analyses of purified GSTs revealed the expression of only the π-class GST in both cell lines. Sulphasalazine inhibited the purified GSTs ($IC_{50}$ of 10 μM for H-69 and 12 μM for H-2496) from both lines in a competitive manner with similar $K_i$ values (6.5 and 7.9 μM for the H-69 and H-2496 cell lines respectively). Cytotoxicity studies revealed that sulphasalazine increased the cytotoxicity of cisplatin towards both cell lines. Isobologram analysis showed that sulphasalazine synergistically enhanced the cytotoxicity of cisplatin towards both cell lines, the magnitude of synergy being remarkably higher in H-69 cells than in H-2496 cells. Our studies indicate that clinically achievable concentrations of sulphasalazine may be useful in modulating cisplatin resistance in malignancies with increased GST-π content.

Cisplatin [*cis*-diamminedichloroplatinum (II); DDP] is an electrophilic platinum coordinate compound which causes DNA damage by forming platinum-DNA coordination complexes (Zwelling & Kohn, 1980). It is one of the most effective antineoplastic agents clinically used against human small-cell lung cancer (SCLC) (Aisner, 1988). However, its clinical efficacy as an antineoplastic agent is curtailed in many cancers owing to inherent or acquired resistance to its cytotoxicity (Loehrer & Einhorn, 1984). Attempts are currently under way to improve the efficacy of DDP by using non-cytotoxic drugs to defeat cellular defence mechanisms that participate in mediating resistance to DDP (Timmer-Bosscha *et al.*, 1992). Cellular defence mechanisms that protect cells from DDP-induced DNA damage (Kelley & Rozencweig, 1989; Perez *et al.*, 1990; Timmer-Bosscha *et al.*, 1992) can be conceptualised as those that repair DDP-induced DNA damage (Teicher *et al.*, 1987; Masuda *et al.*, 1990), those that decrease the accumulation of DDP into the cell (Teicher *et al.*, 1987; Andrews *et al.*, 1988; Bungo *et al.*, 1990) or those that increase the capacity of cytoplasmic defence mechanisms which interfere with the ability of electrophilic toxins, such as DDP, to interact with DNA (Meijer *et al.*, 1990; Kasahara *et al.*, 1991; Muller *et al.*, 1991). Glutathione (GSH), the chief intracellular nucleophile, functions as a scavenger of electrophilic toxins, and its cellular concentrations have been shown to be increased in some DDP-resistant cell lines (Batist *et al.*, 1986; Meijer *et al.*, 1990; Mistry *et al.*, 1991). Glutathione *S*-transferases (GSTs) are multifunctional cellular enzymes which can detoxify electrophiles by conjugating them with GSH (Jakoby, 1978). Increased GST activity has been linked with DDP resistance (Teicher *et al.*, 1987; Saburi *et al.*, 1989; Miyazaki *et al.*, 1990; Puchalski & Fahl, 1990; Sharma *et al.*, 1993). Because GSTs are susceptible to inhibition by a number of non-cytotoxic drugs (van Bladeren & van Ommen, 1991), they are an attractive target for attempts to enhance DDP efficacy using non-cytotoxic inhibitors, such as ethacrynic acid (a non-cytotoxic diuretic drug). Ethacrynic acid has been shown to enhance the cytotoxic effects of certain alkylating agents (Tew *et al.*, 1988). However, the GST-π isoenzyme, which has been linked with malignant transformation and with resistance to alkylating agents (Tsuchida & Sato, 1992), has been suggested to decrease the efficacy of ethacrynic acid in enhancing alkylating agent cytotoxicity (Kuzmich *et al.*, 1992). Furthermore, ethacrynic acid has been found not to enhance the cytotoxicity of DDP towards resistant malignant cell lines (Plumb *et al.*, 1990).

During studies aimed at finding alternative non-cytotoxic GST inhibitors, we have found that sulphasalazine (SS), a drug commonly used for the treatment of inflammatory bowel disorders, is an effective inhibitor of GSTs including GST-π (Ahmad *et al.*, 1992). We have also studied electrophile defence mechanisms, including GSH levels, and activities of enzymes which participate in detoxification of electrophilic toxins, including GSTs, glutathione peroxidase (GPx), glutathione reductase (GR) and glucose-6-phosphate dehydrogenase (G6PD), and have found that among these defence mechanisms GST activity correlated best with degree of DDP resistance in these cell lines (Sharma *et al.*, 1993). The present studies were designed to test the role of GSTs in mediating DDP resistance in two human SCLC cell lines, NCI H-69 and H-2496, in which GST activities parallel DDP resistance, by studying the effect of SS on their GSTs and sensitivity to DDP. The inhibitory effects of SS of GST activity were studied using GSTs purified from these cell lines. A spectrophotometric cytotoxicity assay (Carmichael *et al.*, 1987; Twentyman & Luscombe, 1987) which utilises 3-(4,5-dimethylthiazol-2-yl)-2,5-diphenyltetrazolium bromide (MTT) was used in conjunction with isobologram analysis (Steel & Peckham, 1979) to determine whether SS is able to enhance the cytotoxicity of DDP in a synergistic manner. The major GST isoenzyme in both cell lines was found to be GST-π. SS was found to be a competitive inhibitor of GST-π at concentrations which can be achieved in human serum with its conventional doses (Das & Dubin, 1976). SS was found to enhance the cytotoxicity of DDP in a synergistic manner in both cell lines, but the degree of synergy was considerably greater towards the H-69 cell line, which was relatively more resistant to DDP and which had a higher GST-π content than the H-2496 cell line. These results support the results of our previous studies (Sharma *et al.*, 1993) which have implicated GST-π as a significant determinant of DDP resistance and suggest that SS could be clinically used to enhance the anti-tumour efficacy of DDP in malignant cells overexpressing GST-π.

Correspondence: S. Awasthi.
Received 8 October 1993; and in revised form 3 February 1994.

### Materials and methods

*Reagents and chemicals*

Reagents including SS and 3-(4,5-dimethylthiazol-2-yl)-2,5-diphenyltetrazolium bromide (MTT), 1-chloro-2,4-dinitrobenzene (CDNB), 5,5'-dithiobis-2-nitrobenzoic acid (DTNB), dimethylsulphoxide (DMSO), diethylpyrocarbonate, formamide, guanidinium thiocynate and other reagents were purchased from Sigma (St Louis, MO, USA). A random primed labelling kit was purchased from Boehringer-Mannheim (Indianapolis, IN, USA). Radiolabelled [α-$^{32}$P]dCTP (specific activity $6 \times 10^8$ c.p.m. $\mu g^{-1}$) was purchased from DuPont NEN Products (Billerica, MA, USA). Zetabind nylon membrane was purchased from Cuno Laboratory (Meridien, CT, USA). DDP was obtained from Bristol Laboratories (Evansville, IN, USA). Fetal bovine serum (FBS) was purchased from Intergen Company (Purchase, NY, USA). Cell culture supplies including RPMI-1640 medium, penicillin-streptomycin (P/S) solution and Dulbecco's phosphate-buffered saline (PBS) were purchased from Gibco Laboratories (Grand Island, NY, USA).

*Culture conditions*

Two human SCLC cell lines, NCI H-69 and NCI H-2496, were generous gifts from H. Oie at the National Cancer Institute, Baltimore, MD, USA. These SCLC cell lines, which had never been exposed to chemotherapeutic drugs *in vitro*, grew in suspension cultures in RPMI-1640 medium containing 10% FBS and 1% P/S at 37°C in a 5% carbon dioxide atmosphere. Cells were maintained in the log phase of growth by diluting them 1:3 with medium every 2-3 days.

*Non-protein sulphydryl content of cell lines*

Cells growing in log phase were washed with PBS and homogenate prepared by sonication in 10 mM potassium phosphate buffer, pH 7.0. Non-protein sulphydryl (NPSH) content in the acid-soluble fraction of the homogenate was determined spectrophotometrically using DTNB (Beutler *et al.*, 1963).

*GST activity, purification, Western blot analysis and kinetics of inhibition by sulphasalazine*

GST activity towards CDNB was determined (Habig *et al.*, 1974) on 28,000 *g* supernatants of the homogenate prepared as described above. One unit of GST activity was defined as 1 μmol of dinitrophenyl-*S*-glutathione (Dnp-SG, $\varepsilon_{340} = 9.6\ mM^{-1}\ cm^{-1}$) formed per minute at 25°C. GSTs were purified from cell homogenate of both cell lines using GSH affinity chromatography as used by us previously (Sharma *et al.*, 1993). Western blot analysis on the purified GSTs was performed (Towbin *et al.*, 1979) using polyclonal antibodies specific for the α, μ and π classes of GST. To determine a dose-response curve for inhibition of GST, purified enzyme was incubated with varying concentrations of SS followed by measurement of initial rate of Dnp-SG formation spectrophotometrically. Two concentrations of SS (one above and one below the concentration which caused 50% inhibition of GST activity) were used with varying concentrations of CDNB to determine initial velocity for generating double reciprocal plots. $V_{max}$ and $K_m$ for CDNB and nature of inhibition of GST by SS were determined from these plots. Replots of the slopes from the double reciprocal plots were used to determine $K_i$.

*Northern blot analysis of GST RNA expression in cell lines*

cDNA probes for the α, μ and π class GSTs were prepared by restriction endonuclease digestion of plasmids containing cDNA clones of the α-class GST Ha-1 (a gift from C.P.D. Tu, Department of Molecular and Cell Biology, The Pennsylvania State University, University Park, USA), the π-class GST a gift from M. Muramatsu, Department of Biochemistry, University of Tokyo, Japan) and the μ-class GST (a gift from I. Listowsky, Department of Biochemistry, Albert Einstein Medical College, New York, USA). The cDNA fragments were purified by a method utilising low melting temperature agarose gel electrophoresis (Falson, 1992). The probes were labelled by the random-primed labelling method according to the manufacturer's (Boehringer-Mannheim) recommendations using [α-$^{32}$P]dCTP, and the unincorporated nucleotides were removed by filtration through Millipore Ultra Free MC filter units [10,000 nominal molecular weight limit (NMWL)]. RNA was isolated from $4 \times 10^8$ cells by acid guanidinium thiocyanate-phenol-chloroform extraction (Chomoczynski & Sacchi, 1987) and the concentration and purity were determined spectrophotometrically by measuring absorbance at 260 and 280 nm. A 25 μg aliquot of the isolated RNA from both cell lines was subjected to formaldehyde-agarose gel electrophoresis in a 1.2% agarose gel at 35 V overnight and transblotted to Zetabind nylon membrane. Nylon filters were prehybridised overnight at 50°C in hybridisation buffer (0.2 M sodium phosphate pH 7.2, 1% bovine serum albumin, 7% SDS, 1 mM EDTA and 15% formamide) and hybridised in the same buffer with the corresponding $^{32}$P-labelled DNA probes at 50°C overnight. Filters were washed once at 50°C for 25 min, and twice at room temperature for 5 min each in washing buffer (40 mM sodium phosphate pH 7.2, 1 mM EDTA and 1% SDS). Filters were exposed to Kodak XAR-5 X-ray film at −80°C using intensifying screens.

*Determination of $IC_{50}$ for cisplatin and SS*

A modified MTT cytotoxicity assay similar to that previously described (Carmichael *et al.*, 1987; Twentyman & Luscombe, 1987) was standardised to determine the numbers of viable cells after cisplatin and/or SS treatments. Briefly, aliquots containing approximately 5,000 cells were taken from flasks containing cells in log phase growth at a density of $3-5 \times 10^5$ cells $ml^{-1}$ and inoculated into 96-well plates. The cells were diluted with medium containing various concentrations of DDP and/or SS. After 72 h, 20 μl of MTT (2 mg $ml^{-1}$) was added to each well followed by incubation for 1 h at 37°C. The cells were centrifuged in Eppendorf tubes at 28,000 *g* for 10 min. The supernatant was discarded and the pellet thoroughly solubilised in 50 μl of DMSO, followed by addition of 1 ml of isopropanol. The absorption of this extract was recorded at 560 nm using a Gilford Response spectrophotometer. Surviving cell number was quantified using a standard curve of cell number versus absorbance at 560 nm. The $IC_{50}$ values for DDP and SS were determined from a plot of percentage surviving cells (compared with control cells) versus drug concentrations. All cytotoxicity experiments were conducted four times, each with triplicate determinations, and the average values and standard deviations were used to construct log survival curves and isobolograms.

*Isobologram analysis*

Isobolograms and envelopes of additivity were constructed according to the previously described method (Steel & Peckham, 1979). Briefly, the $IC_{50}$ values obtained for DDP and SS alone were plotted on a Cartesian plane at equal distances from the origin on the *x*- and *y*-axes respectively. The line connecting these points was designated the theoretical isotoxic dose line, or the combination of DDP and SS concentrations at which 50% of cells would survive provided the log survival curves of both drugs were linear. However, this theoretical isotoxic dose line, which represents simple additivity of cytotoxicity of both drugs at any combination of concentrations, may not apply to non-linear log survival curves of one or both drugs. Since, the survival curve for SS was not linear, an envelope of additivity of DDP and SS was calculated from the cell survival data as described previously (Steel & Peckham, 1979). The $IC_{50}$ values of DDP determined

**Figure 1** Western blot analysis of GSTs purified from human SCLC cell lines, H-69 and H-2496, with polyclonal antibodies specific for GST-α, -μ and -π. In all three panels, lane 1 contained purified antigens as positive controls. Lanes 2 and 3 contained 5 μg of GSH affinity-purified GSTs from the H-69 and H-2496 cell lines respectively.

at several fixed concentrations of SS (below the $IC_{50}$ value of SS) were plotted against the corresponding concentration of SS. A curve thus generated and lying below the envelope of additivity was taken as the evidence of synergy between the cytotoxic effects of two drugs.

### Results and discussion

The average NPSH contents of the H-69 and H-2496 cell lines from three separate determinations were found to be $60.4 \pm 5.8$ and $44 \pm 7.2$ nmol $mg^{-1}$ protein respectively. One hour after exposure to 1 mM SS, average NPSH contents of the H-69 and H-2496 cell lines from three separate determinations ($62.2 \pm 6.4$ and $41.0 \pm 5.8$ nmol $mg^{-1}$ protein respectively) were not significantly altered acutely by exposure to SS. These results are consistent with a previous report that SS is not conjugated with GSH (Das & Dubin, 1976) and suggest that any effect of SS on DDP sensitivity is not caused by SS-induced changes in NPSH. Western blot analysis of purified GSTs revealed the presence of only the GST-π isoenzyme in these cell lines (Figure 1). The results of Northern blot analysis revealed that only GST-π was present in both cell lines, and GST-α or -μ RNA was not detected (figures not presented). These results were consistent with previous studies on the composition of GST isoenzymes in human SCLC cell lines (Miyazaki *et al.*, 1990). The average GST activity, determined in homogenates of the H-69 and H-2496 cell lines in three separate experiments ($317 \pm 7$ *vs* $9 \pm 1$ mU $mg^{-1}$ protein respectively), indicated that the H-69 cell line contained 34-fold higher GST activity than the H-2496 cell line (Sharma *et al.*, 1993). SS inhibited GSTs of both cell lines with 50% inhibition of activity towards CDNB as seen at 10 and 12 μM for the H-69 and H-2496 cell lines respectively (Figure 2). SS was found to be a competitive inhibitor of the GSTs isolated from both cell lines, with $K_i$ values of 6.5 and 7.9 μM for the H-69 and H-2496 cell lines respectively.

An MTT cytotoxicity assay was used to determine the sensitivity of the SCLC cell lines to DDP and sulphasalazine alone and in combination. This assay is based on the optical measurement of a formazan dye which is cleaved from MTT by mitochondrial dehydrogenase of actively respiring mitochondria and has been shown to correlate with results of colony-forming cytotoxicity assays in SCLC cell lines (Carmichael *et al.*, 1987). The calibration curve of the MTT cytotoxicity assay revealed that absorbances at 560 nm were linear in the range 0.05–1.0 with respect to cell number determined by counting disaggregated cells in a haemocytometer (data not presented). Using this assay, the H-69 cell line was found to be approximately 6-fold more resistant to DDP than the H-2496 cell line (average $IC_{50}$ values from four separate experiments of $25 \pm 3.9$ and $4.5 \pm 1.0$ μM for the H-69 and H-2496 lines respectively). The log survival curves for the H-69 and H-2496 cell lines for DDP (Figure 3a) and SS alone (Figure 3b) are presented. These survival curves were linear with respect to DDP concentrations, but non-linear with respect to SS concentrations, for both cell lines. On the basis of these results, the use of envelopes of additivity became necessary for the analysis of synergy in isobolograms rather than the theoretical isotoxic dose line, which represented simple additivity of cytotoxicity between the two drugs. Isobolograms showing the theoretical envelopes of additivity and the actual isotoxic dose curves for combinations of SS and DDP are shown for the H-69 (Figure 4a) and H-2496 (Figure 4b) cell lines respectively. The isotoxic dose curves for both cell lines were found to lie below the theoretical isotoxic dose line, indicating that SS was able to enhance the cytotoxicity of DDP in a synergistic manner. The degree of synergy, reflected in the degree of deviation from the envelope of additivity, was significantly greater for the more resistant H-69 cell line having higher GST activity than the H-2496 cell line. Greater synergy between SS and DDP in the H-69 cell line, which contains significantly higher GST activity than the DDP-sensitive H-2496 cell line, suggested that GST may play a prominent role in mediating DDP resistance. Since GSTs have not been

**Figure 2** Dose-response curve for inhibition of purified GSTs by sulphasalazine. The percentage inhibition, with respect to control, of activity of GSTs purified from the H-69 (●) and H-2496 (O) towards CDNB at each concentration of sulphasalazine is plotted against the corresponding sulphasalazine concentration. The values presented represent means and standard deviations from three separate determinations.

**Figure 3** Log survival curves of the H-69 (+) and H-2496 (●) cell lines with respect to (**a**) cisplatin and (**b**) sulphasalazine concentrations. The results presented are mean and standard deviations calculated from triplicate determinations in four separate experiments.

shown to catalyse the conjugation of GSH with DDP, further studies are required to elucidate the mechanisms through which GSTs may participate in defence of cells towards DDP and define the mechanism of enhancement of DDP cytotoxicity by SS. In addition to GST inhibition, SS has been shown to affect the synthesis of leukotrienes and prostaglandins from arachidonic acid contained in plasma membranes (Tornhamre *et al.*, 1989), but its effects on protein kinase C have not been reported. Since inhibitors of protein kinase C can potentiate DDP cytotoxicity (Timmer-Bosscha *et al.*, 1992), future studies on the effect of sulphasalazine on protein kinase C activity may be helpful in delineating an additional mechanism for the observed enhancement of DDP cytotoxicity by SS.

**Figure 4** Isobolograms of cisplatin and sulphasalazine (——) with the envelopes of additivity (---) for the (**a**) H-69 and (**b**) H-2496 cell lines. The isobolograms and envelopes of additivity were constructed using the method described by Steel & Peckham (1979). The values presented represent means and standard deviations from triplicate determinations of $IC_{50}$ values in four separate determinations.

Our studies suggest that SS should be added to the long list of agents which can enhance DDP cytotoxicity (Timmer-Bosscha *et al.*, 1992). The marked degree of enhancement of DDP toxicity in the H-69 cell line at the lower concentrations of SS is interesting. Based on SS pharmacokinetics in humans, concentrations of SS up to 25 μM can be achieved in human plasma at relatively non-toxic doses of SS (Das & Dubin, 1976). Furthermore, animal pharmacokinetic studies of SS show that its concentrations in lung and connective tissues are significantly higher (in the range of 0.5 mM) than in serum, and they can persist for prolonged periods after bolus doses of SS (Hanngren *et al.*, 1963). These findings can be helpful in designing chemotherapeutic regimens to test the efficacy of SS in modulating the anti-cancer efficacy of DDP in human malignancy, particularly lung cancer.

This work was supported in part by Grant GM-32304 awarded by the National Institute of General Medical Sciences (to Y.C.A.). S.A. wishes to express thanks to Don W. Powell, MD, Chair, Department of Internal Medicine, University of Texas Medical Branch, for providing funds for these studies. The secretarial assistance of Mrs Alicia Woods is acknowledged.

### References

AHMAD, H., SINGHAL, S.S. & AWASTHI, S. (1992). The inhibition of α, μ, and π class isozymes of glutathione S-transferases by sulfasalazine, 5-aminosalicylic acid and sulfapyridine. *Biochem. Arch.*, **8**, 355-361.

ANDREWS, P.A., VELURY, S., MANN, S.C. & HOWELL, S.B. (1988). *cis*-Diamminedichloroplatinum(II) accumulation in sensitive and resistant human ovarian carcinoma cells. *Cancer Res.*, **48**, 68-73.

AISNER, J. (1988). Chemotherapy for small cell carcinoma of the lung. In *Lung Cancer - A Comprehensive Treatise*, Bitran, J.D., Golomb, H.M., Little, A.G. & Weichselbaum, R.R. (eds) pp. 307-327. Grune & Stratton: New York.

BATIST, G., BEHRENS, B.C., MAKUCH, R.L., HAMILTON, T.C., KATKI, A.G., LOUIE, K.G., MEYERS, C.E. & OZOLS, R.F. (1986). Serial determinations of glutathione levels and glutathione-related enzyme activities in human tumor cells *in vitro*. *Biochem. Pharmacol.*, **35**, 2257-2259.

BEUTLER, E., DURON, O. & KELLY, B.M. (1963). Improved method for the determination of blood glutathione. *J. Lab. Clin. Med.*, **61**, 882-888.

BUNGO, M., FUJIWARA, Y., KASAHARA, K., NAKAGAWA, K. OHE, Y., SASAKI, Y., IRINO, S. & SAIJO, N. (1990). Decreased accumulation as a mechanism of resistance to *cis*-diamminedichloroplatinum(II) in human non-small cell lung cancer cell lines: relation to DNA damage and repair. *Cancer Res.*, **50**, 2549-2553.

CARMICHAEL, J., DEGRAFF, W.G., GAZDAR, A.F., MINNA, J.D. & MITCHELL, J.B. (1987). Evaluation of a tetrazolium-based semiautomated colorimetric assay: assessment of chemosensitivity testing. *Cancer Res.*, **47**, 936-942.

CHOMOCZYNSKI, P. & SACCHI, N. (1987). Single step method of RNA isolation by acid guanidinium thiocyanate-phenol-chloroform extraction. *Anal. Biochem.*, **162**, 156-159.

DAS, K.M. & DUBIN, R. (1976). Clinical pharmacokinetics of sulfasalazine. *Clin. Pharmacol.*, **1**, 406-425.

FALSON, P. (1992). Improved phenol-based method for the isolation of DNA fragments from low melting temperature agarose gel. *Biotechniques*, **13**, 22-26.

HABIG, W.H., PABST, M.J. & JAKOBY, W.B. (1974). Glutathione S-transferases: the first enzymatic step in mercapturic acid formation. *J. Biol. Chem.*, **249**, 7130-7139.

HANNGREN, A., HANSSON, E., SVARTZ, N. & ULLBERG, S. (1963). Distribution and metabolism of salicyl-azo-sulfapyridine. I. A study with $C^{14}$-salicyl-azo-sulfapyridine and $C^{14}$-5-amino-salicylic acid. *Acta Med. Scand.*, **173**, 61-72.

JAKOBY, W.B. (1978). The glutathione S-transferases: a group of multifunctional detoxification proteins. *Adv. Enzymol.*, **46**, 383-414.

KASAHARA, K., FUJIWARA, Y., NISHIO, K., OHMORI, T., SUGIMOTO, Y., KOMIYA, K., MATSUDA, T. & SAIJO, N. (1991). Metallothionein content correlates with the sensitivity of human small cell lung cancer cell lines to cisplatin. *Cancer Res.*, **51**, 3237-3242.

KELLEY, S.L. & ROZENCWEIG, M. (1989). Resistance to platinum compounds: mechanisms and beyond. *Eur. J. Cancer Clin. Oncol.*, **25**, 1135-1140.

KUZMICH, S., VANDERVEER, L.A., WALSH, W.S., LACRETA, F.P. & TEW, K.D. (1992). Increased levels of glutathione S-transferase-π as a mechanism of resistance to ethacrynic acid. *Biochem. J.*, **281**, 219-224.

LOEHRER, P.J. & EINHORN, L.H. (1984). Diagnosis and treatment - drugs five years later: cisplatin. *Ann. Intern. Med.*, **100**, 704-713.

MASUDA, H., TANAKA, T., MATSUDA, H. & KUSABA, I. (1990). Increased removal of DNA-bound platinum in a human ovarian cancer cell line resistant to *cis*-diamminedichloro-platinum(II). *Cancer Res.*, **50**, 1863-1866.

MEIJER, C., MULDER, N.H., HOSPERS, G.A.P, UGES, D.R.A. & DE VRIES, E.G.E. (1990). The role of glutathione in resistance to cisplatin in human small cell lung cancer cell lines. *Br. J. Cancer*, **62**, 72-77.

MISTRY, P., KELLAND, L.R., ABEL, G., SIDHAR, S. & HARRAP, K.R. (1991). The relationship between glutathione, glutathione S-transferase and cytotoxicity of platinum drugs and melphalan in eight human ovarian carcinoma cell lines. *Br. J. Cancer*, **64**, 215-220.

MIYAZAKI, M., KOHNO, K., SABURI, Y., MATSUO, K., ONO, M., KUWANO, M., TSUCHIDA, S., SATO, K., SAKAI, M. & MURAMATSU, M. (1990). Drug resistance to cis-diamminedichloroplatinum (II) in chinese hamster ovary cell lines transfected with glutathione S-transferase pi gene. *Biochem. Biophys. Res. Commun.*, **166**, 1358-1364.

MULLER, M.R., WRIGHT, K.A. & TWENTYMAN, P.R. (1991). Differential properties of cisplatin and tetraplatin with respect to cytotoxicity and perturbation of glutathione levels. *Cancer Chemother. Pharmacol.*, **28**, 273-276.

PEREZ, R.P., HAMILTON, T.C. & OZOLS, R.F. (1990). Resistance to alkylating agents and cisplatin: insights from ovarian carcinoma model systems. *Pharmacol. Ther.*, **48**, 19-27.

PLUMB, J.A., MILROY, R., BICKNELL, S.R. & KAY, S.B. (1990). Glutathione S-transferase, P-glycoprotein and drug resistance in small cell lung cancer cell lines. *Proc. Am. Assoc. Cancer Res.*, **31**, 369.

PUCHALSKI, R.B. & FAHL, W.E. (1990). Expression of recombinant glutathione S-transferase π, Ya or $Yb_1$ confers resistance to alkylating agents. *Proc. Natl Acad. Sci. USA*, **87**, 2443-2447.

SABURI, Y., NAKAGAWA, M., ONO, M., SAKAI, M., MURAMATSU, M., KOHNO, K. & KUWANO, M. (1989). Increased expression of glutathione S-transferase gene in *cis*-diammine-dichloroplatinum(II)-resistant variants of chinese hamster ovary cell lines. *Cancer Res.*, **49**, 7020-7025.

SHARMA, R., SINGHAL, S.S., SRIVASTAVA, S.K., BAJPAI, K.K., FRENKEL, E.P. & AWASTHI, S. (1993). Glutathione and glutathione linked enzymes in human small cell lung cancer cell lines. *Cancer Lett.*, **75**, 111-119.

STEEL, G.G. & PECKHAM, M.J. (1979). Exploitable mechanisms in combined radiotherapy-chemotherapy: the concept of additivity. *Int. J. Rad. Oncol. Biol. Phys.*, **5**, 85-91.

TEICHER, B.A., HOLDEN, S.A., KELLEY, M.J., SHEA, T.C., CUCCHI, C.A., ROSOWSKY, A., HENNER, W.D. & FREI, III, E. (1987). Characterization of a human squamous carcinoma cell line resistant to cis-diamminedichloroplatinum (II). *Cancer Res.*, **47**, 388-393.

TEW, K.D., BOMBER, A.M. & HOFFMAN, S.J. (1988). Ethacrynic acid and piriprost as enhancers of cytotoxicity in drug resistant and sensitive cell lines. *Cancer Res.*, **48**, 3622-3625.

TIMMER-BOSSCHA, H., MULDER, N.H. & DE VRIES, E.G.E. (1992). Modulation of cis-diamminedichloroplatinum (II) resistance: a review. *Br. J. Cancer*, **66**, 227-238.

TORNHAMRE, S., EDENIUS, C., SMEDGARD, G., BIRGITTA, S. & LINDGREN, J.A. (1989). Effects of sulfasalazine and a sulfasalazine analogue on the formation of lipooxygenase and cyclooxygenase products. *Eur. J. Pharmacol.*, **169**, 225-234.

TOWBIN, H., STAEHELIN, T. & GORDON, J. (1979). Electrophoretic transfer of proteins from polyacrylamide gels to nitrocellulose sheets: procedure and some applications. *Proc. Natl Acad. Sci. USA*, **76**, 4350-4354.

TSUCHIDA, S. & SATO, K. (1992). Glutathione transferases and cancer. *Crit. Rev. Biochem. Mol. Biol.*, **27**, 337-384.

TWENTYMAN, P.R. & LUSCOMBE, M. (1987). A study of some variables in a tetrazolium dye (MTT) based assay for cell growth and chemosensitivity. *Br. J. Cancer*, **56**, 279-285.

VAN BLADEREN, P.J. & VAN OMMEN, B. (1991). The inhibition of glutathione S-transferases: mechanisms, toxic consequences and therapeutic benefits. *Pharmacol. Ther.*, **51**, 35-46.

ZWELLING, L.A. & KOHN, K.W. (1980). Effects of cisplatin on DNA and the possible relationships to cytotoxicity and mutagenicity in mammalian cells. In: *Cisplatin: Current Status and New Developments*, Prestayko, A., Crooke, S. & Carter, S. (eds) pp. 21-37. Academic Press: New York.